# Somatic mutations of thymic epithelial tumors with myasthenia gravis

**DOI:** 10.3389/fonc.2023.1224491

**Published:** 2023-08-21

**Authors:** Eleonora Pardini, Federico Cucchiara, Sara Palumbo, Giulia Tarrini, Alessia Di Vita, Fabio Coppedè, Vanessa Nicolì, Melania Guida, Michelangelo Maestri, Roberta Ricciardi, Vittorio Aprile, Marcello C. Ambrogi, Serena Barachini, Marco Lucchi, Iacopo Petrini

**Affiliations:** ^1^Department of Translational Research and of New Surgical and Medical Technologies, University of Pisa, Pisa, Italy; ^2^Department of Clinical and Experimental Medicine, University of Pisa, Pisa, Italy; ^3^Medical Genetics, Department of Translational Research and of New Surgical and Medical Technologies, University of Pisa, Pisa, Italy; ^4^Neurology Unit, Department of Clinical and Experimental Medicine, University of Pisa, Pisa, Italy; ^5^Thoracic Surgery, Department of Surgical, Medical and Molecular Pathology and Critical Care, University of Pisa, Pisa, Italy

**Keywords:** GTF2I, thymoma, next-generation sequencing, myasthenia gravis, thymic epithelial tumors

## Abstract

**Background:**

Thymic epithelial tumors are rare malignant neoplasms that are frequently associated with paraneoplastic syndromes, especially myasthenia gravis. GTF2I is an oncogene mutated in a subgroup of thymomas that is reputed to drive their growth. However, for GTF2I wild-type tumors, the relevant mutations remain to be identified.

**Methods:**

We performed a meta-analysis and identified 4,208 mutations in 339 patients. We defined a panel of 63 genes frequently mutated in thymic epithelial tumors, which we used to design a custom assay for next-generation sequencing. We sequenced tumor DNA from 67 thymomas of patients with myasthenia gravis who underwent resection in our institution.

**Results:**

Among the 67 thymomas, there were 238 mutations, 83 of which were in coding sequences. There were 14 GTF2I mutations in 6 A, 5 AB, 2 B2 thymomas, and one in a thymoma with unspecified histology. No other oncogenes showed recurrent mutations, while sixteen tumor suppressor genes were predicted to be inactivated. Even with a dedicated assay for the identification of specific somatic mutations in thymic epithelial tumors, only GTF2I mutations were found to be significantly recurrent.

**Conclusion:**

Our evaluation provides insights into the mutational landscape of thymic epithelial tumors, identifies recurrent mutations in different histotypes, and describes the design and implementation of a custom panel for targeted resequencing. These findings contribute to a better understanding of the genetic basis of thymic epithelial tumors and may have implications for future research and treatment strategies.

## Introduction

Thymic epithelial tumors (TETs) are uncommon malignant neoplasms with an incidence of approximately 0.32 per 100,000 people/year (1). According to the 2020 classification of the World Health Organization, TETs are divided into thymomas and thymic carcinomas. Thymomas account for about 90% of TETs and are further classified into A, AB, B1, B2, and B3 histotypes depending on their morphological features (2). About 40% of thymomas show myasthenia gravis, a paraneoplastic autoimmune syndrome, which is absent in thymic carcinomas and micronodular thymomas (3). Myasthenia gravis is more common in type B than type A and AB thymomas (4). Historically, tumor stage has been defined according to the Masaoka and Koga system, but more recently, it is determined according to the eighth edition of TNM (5). Surgery is the cornerstone treatment for localized disease, ensuring definitive tumor eradication in most stage I and II TETs (6). Chemotherapy with schedules containing cisplatin and anthracyclines is effective in TETs and can be used in the neoadjuvant setting to bring back to resectability locally advanced stage III tumors (7). While hematogenous or lymphatic metastases are common in thymic carcinomas, thymomas frequently grow through local infiltration, and their most frequent way of diffusion is through pleural metastases (2). Several phase II trials investigated the efficacy of chemotherapy for the first-line treatment of metastatic TETs, showing an objective response in 50-90% of cases (8). Unfortunately, after disease progression, further lines of chemotherapy become much less effective, and there is an urgent need for novel treatments. Even if GTF2I mutations are common in A and AB thymomas, there are no clinically relevant targets in TETs except for KIT mutations observed in only 10% of thymic carcinomas (9). Nevertheless, targeted therapies such as sunitinib and lenvatinib have shown good efficacy in pretreated thymic carcinomas, with an objective response rate of 26% and 38%, respectively (10, 11). Immunotherapy is effective in TETs but severely increases the risk of autoimmune-mediated complications, especially in thymomas, and therefore can be considered only in some cases of thymic carcinomas (12, 13).

Several attempts to identify somatic mutations that characterize TETs have been made in recent years, including whole-genome sequencing of two cases, exome sequencing both from our group and from The Cancer Genome Atlas (TCGA), and targeted resequencing using panels of genes frequently mutated in other kinds of cancers (4, 9, 14–21). While somatic mutations are usually observed in thymic carcinomas, thymomas present only occasional mutations, with the exception of GTF2I, which is common in A and AB thymomas. Reviewing the literature, we defined a panel of genes frequently mutated in TETs and performed targeted resequencing of a series of surgically resected thymomas to favor the identification of mutations in this subset of tumors.

## Materials and methods

### Sample selection

Tumor samples were collected during the surgical resection of thymomas, either thymectomy or resection of pleural metastases, at Pisa University Hospital. All patients included in the study had myasthenia Gravis with anti-acetylcholine receptor antibodies, and therefore, thymic carcinomas were not included in this evaluation. The histotype of thymoma was defined according to 2015 World health organization (WHO) classification, and the stage was determined according to the Masaoka and Koga system. Patients gave informed written consent for inclusion in the study, which was conducted in accordance with the Declaration of Helsinki and approved by the Ethics Committee of Tuscany Region for Clinical Trials - Section of North West Area (CEAVNO) (Protocol number 21302/2015).

### Nucleic acid extraction

Tumor samples were snap frozen at -80°C. DNA was extracted using the QIAmp DNA Mini Kit (Qiagen, Hilden, Germany) following the manufacturer’s protocol. DNA was quantified firstly with Nanodrop (Thermo Scientific™, Waltham, MA, USA) to evaluate ethanol and protein contamination and subsequently with Qubit BR Assay (Thermo Scientific™).

### Next-generation sequencing

We designed a custom panel for targeted resequencing of frequently mutated genes in thymic epithelial tumors using Suredesign (Agilent Technologies, Palo Alto, CA, USA). The genes were selected from a review of the literature, and a detailed description of the selection is provided in the results section. Following the vendor instructions, libraries of target enriched genes were prepared using the Halopex HS system (Agilent Technologies) and 100 ng of tumor DNA. Pair-end reads of 151 nucleotides were generated using an Illumina-V2 cartridge for the MiSeq sequencer (Illumina, San Diego, CA, USA). Five samples were pooled in the same flow cell. For a subset of 5 samples with *GTF2I* mutation, results were confirmed by sequencing tumor DNA using a targeted enrichment custom kit (Illumina) implemented using Design studio (Illumina) for the same panel of genes.

### Bioinformatic analysis of sequencing data and Interpretation

First, we used FastQCv011.9 (22) (http://www.bioinformatics.babraham.ac.uk/projects/fastqc) and Fastp v0.20.1 (23) to perform quality profiling, adapter trimming, reads filtering, reads pruning and polyG/polyX trimming on fastq files from sequencing systems.

Then paired-end reads were mapped to GRCh37/hg19, using the BWA-MEM alignment algorithm (https://arxiv.org/abs/1303.3997).

The CleanSam tool soft-clipped the mapped reads beyond-end-of-reference alignments while setting the mapping quality (MAPQ) value to 0 for unmapped reads (http://broadinstitute.github.io/picard, accessed on 08 December 2021). The FixMateInformation tool verified mate-pair information between mates and fixed it when needed (http://broadinstitute.github.io/picard, accessed on 08 December 2021). Finally, reads were sorted by SAMtools (24).

Coverage of the genomic regions, annotated in the custom-made sequencing panel (**Supplementary Tables 4** and **5**) had an average of 1247.96 reads. An extensive report of the sequencing metrics is provided in **Supplementary Table 6**.

Sequence alignment data were reported in binary alignment map (BAM) files and processed using the Genome Analysis Tool Kit (GATK) (25) pipeline to remove low mapping quality reads (defined by a MAPQ < 20) and realign the genomic regions around potential insertions/deletions (indels). Base quality scores were recalibrated for the BAM files using GATK.

Somatic single-nucleotide variants (SNVs) and indels were identified by using VarDict (26), variant calling algorithms in *tumor-only* mode.

We excluded rare variants reported in the 1000 Genomes Project (27), and/or in the non-cancer database gnomAD v3 (28) with allele frequency ≥0.01% (29, 30).

To remove diluted variants, SNVs and indels were further filtered and accepted if the following requirements were met: 1) there were at least 20 reads covering the mutation locus in the tumor BAM file; 2) there were at least 4% of reads carrying the mutation in the tumor BAM file. These parameters were chosen by comparing the results of the five samples sequenced using both the Agilent Haloplex and the Illumina DNA enrichment platforms to optimize the concordance between the two assays.

To filter out germline variations, in the absence of sequencing results of non-tumoral DNA from the patients, we removed SNV calls candidate to be homozygous (allele fraction 100%) and heterozygous (alle fraction 50%) polymorphisms. Since we expect tumor somatic mutations to be diluted in normal DNA, we excluded mutations with allele fraction ≥45%, Phred-scaled Fisher’s exact test for strand bias >60 and/or strand bias odds ratio >3, mean mismatches in reads ≥3, signal to noise ratio <4, and reported status of microsatellite instability for the investigated genomic loci. GTF2I mutations were used as an internal control, showing an allele fraction between 4% and 22% in our tumor samples. GTF2I mutations are common in A thymomas, which are poor of thymocytes and rich in tumor cells.

Somatic mutations of the coding regions identified were further annotated using Annovar (31).

The frequency and type of mutations were investigated using the R package MAFtools (32). The package also allowed us to extract mutational signatures as functions of specific patterns of nucleotide substitutions (33) and compared them with those validated in the *Catalogue Of Somatic Mutations In Cancer* (COSMIC; cancer.sanger.ac.uk) (34).

## Results

### Metanalysis of published mutations in thymic epithelial tumors

**Figure 1:**
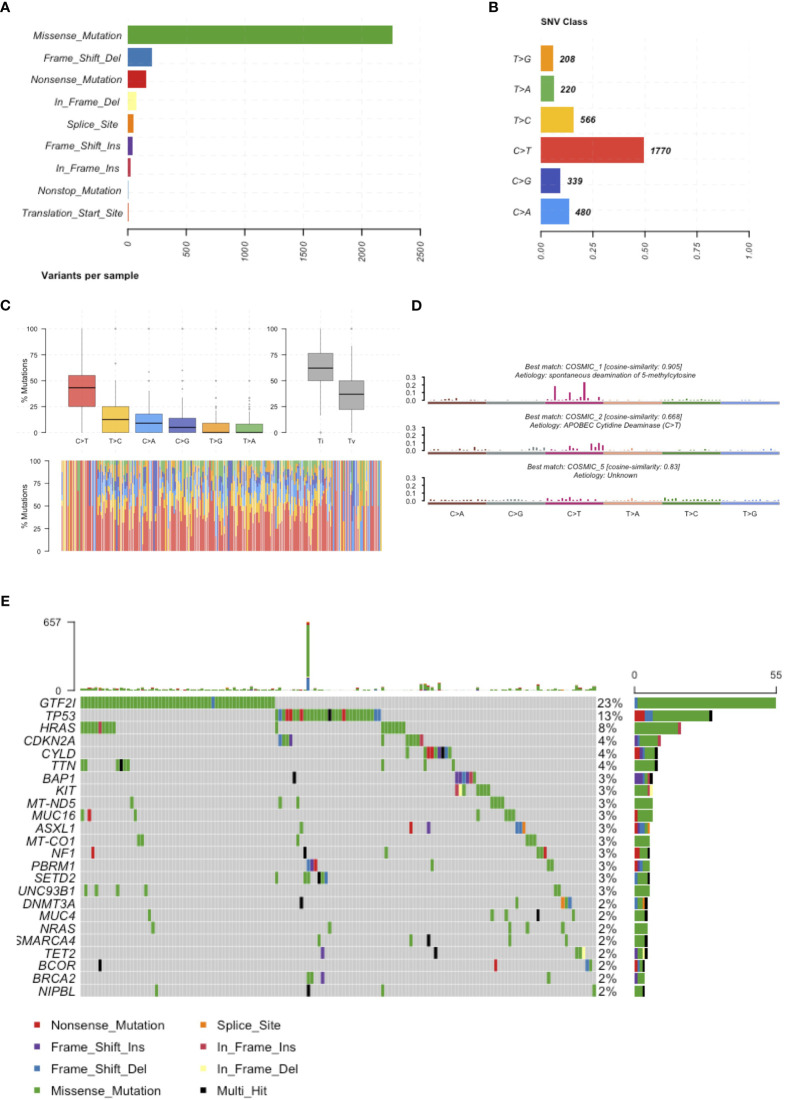
Mutations reported in the literature. **(A)** Number of mutations affecting the coding sequence classified according to their effect on the protein. **(B)** Frequency of single nucleotide substitutions. **(C)** Boxplot representation of mutations observed in the coding sequence of TETs in the upper representation and in each single patients in the lower representation. **(D)** Mutational signatures enriched in TETs. **(E)** Most frequently mutated genes in TETs and type of mutation observed.

In February 2018, we reviewed the scientific literature reporting genomic profiling obtained by next-generation sequencing (NGS) of TETs available in PubMed. We identified 9 trials (**Table 1**) that included data from 339 patients and described 4208 mutations. Multiple technologies of NGS have been adopted, including whole-genome sequencing, exome sequencing, and targeted resequencing of different panels of “cancer-related” genes (range: 50-409 genes). All identified mutations were pooled together (**Supplementary Table 1**) and annotated using Annovar (31) according to UCSC hg19, except for mitochondrial genes that have been aligned to NCBI build-38 reference genome (**Supplementary Table 2**). We selected mutations of the coding sequences (including splicing site mutations) with a non-synonymous effect on protein translation. Point mutations were the most observed, with a dramatic prevalence of missense mutations (**Figure 1A**). The most common point mutations were C to T transitions (**Figures 1B, C**). The mutational signature enriched for TETs were spontaneous deamination of 5’-methylcytosine, APOBEC cytidine deaminase, and a signature of unknown etiology (**Figure 1D**). We identified 781 mutations affecting the same genes in at least 2 different samples (**Supplementary Table 3**). Our list of recurrent mutations in TETs included 266 genes. The most common mutated genes were GTF2I (55 patients), TP53 (31 patients), HRAS (18 patients), TTN (11 patients), BAP1, CDKN2A, and CYLD (10 patients). There were 205 genes with mutations in only 2 patients (**Figure 1C**). A thymic carcinoma with a nonsense mutation in the mismatch repair gene MLH1 E37* had a significantly higher number of mutations: 936 (**Figure 1E**). In line with previous reports (4, 9) different histotypes of TETs harbor different mutated genes. Indeed, from the co-occurrence table, we observed GTF2I mutations together with HRAS, TTN, and UNC93B1 mutations. GTF2I was mutually exclusive with respect to TP53 mutations. GTF2I is more frequently mutated in A and AB thymomas, whereas TP53 in thymic carcinomas and B3 thymomas. A trend for the absence of co-occurrence with GTF2I was observed also for CDKN2A, BAP1, CYLD, and KIT mutations, which are genes frequently mutated in B3 thymomas and thymic carcinomas. Mutations of BRCA2, SETD2, PBRM1, and CDKN2A significantly co-occurred with TP53 (**Figure 1D**). These data further support the presence of at least two molecularly distinct types of TETs. Tumors with GTF2I and TP53 mutations have a similar pattern of mutations, with C>T transitions being the most common events and predominant in the missense mutations (**Supplementary Figures S1** and **S2**). The signature enriched in GTF2I mutant tumors were COSMIC 1: spontaneous deamination of 5-methylcytosine and COSMIC 5: unknown etiology, whereas in TP53 mutant tumors, COSMIC 1: spontaneous deamination of 5-methylcytosine and COSMIC 6: defective mismatch repair (even if the thymic carcinoma with microsatellite instability was removed from the analysis; **Supplementary Figures S3** and **S4**).

**Table 1:** Summary of reports evaluating somatic mutations in TET using Next-Generation Sequencing (NGS).

Authors	Journal	Year	TETs	Mutations	NGS technology	
R Belani, et al. (14)	Oncogenesis	2014	1	17	Whole Genome Sequencing
F. Enkner, et al. (15)	Pathology and Oncology Research.	2016	72	27	Ion AmpliSeq Cancer Hotspot Panel v2 (50 cancer genes)
J. Wheler, et al. (16)	Oncotarget	2013	7	4	Targeted Resequencing 182 Cancer Related Genes (Foundation Medicine)
I. Petrini, et al. (17)	Plos One	2013	1	12	Whole Genome Sequencing
I. Petrini, et al. (9)	Nature Genetics	2014	42	836	Whole Exome Sequencing
M. Shitara, et al. (21)	Lung Cancer	2014	12	25	Targeted Resequencing 409 Cancer Related Genes
Y. Li, et al. (19)	European Journal of Endocrinology	2017	9	137*	Whole Exome Sequencing
Y. Wang, et al. (20)	Scientific Reports	2014	78	86	Targeted Resequencing of 197 Cancer Related Genes
M. Radovich, et al. (4)	Cancer Cell	2018	117	3064	Whole Exome Sequencing

*Only three mutations out of 137 were included in our analysis because coordinates were available.

### Design of a custom panel for the identification of somatic mutations of thymic epithelial tumor

In our panel, we included genes that are possibly relevant for TET growth based on two criteria: 1) genes with mutations in more than five patients and 2) genes known to be proto-oncogenes or tumor suppressor genes if found mutated in two or more patients. Among the genes mutated in more than five patients, TTN, MUC4, MUC16, and UNC93B1 were excluded from our panel. *TTN* is the longest gene present in the genome (109224 bp of isoform NM_001267550 transcript) and is frequently mutated in NGS trials because of the probability to detect a mutation increases along with the increase of the number of bases of the gene. *MUC4* and *MUC16* belong to the family of mucins and were unlikely to be responsible for TET growth. Moreover, mutations affecting the *MUC4* sequence are included in a region where alternative alignments of reads have been described (35, 36). However, mutations of *MUC4* and *MUC16* are described predominantly in TET histotypes rich in cancer cells and not in thymocyte-rich tumors, suggesting the actual presence of the mutation. The mutations were not previously described in COSMIC and were not predicted to alter the protein structure and function (**Supplementary Table 2**, MCAP13 range 0.000677-0.006565). UNC93B1 was mutated in six tumors, and in five cases, the mutation was chr11:67759316C/T, a known frequent polymorphism (PF=0.063) without a predicted effect on protein structure and function. Our panel of genes was composed of 63 genes identified from our review of TET literature, to which we added 14 genes known to be frequently mutated in cancer. For known tumor suppressor genes, we included the entire coding sequence, whereas for oncogenes, we sequenced only frequently mutated hotspots, unless we observed mutations in a different part of the coding sequence in published sequencing reports of TETs. To sequence the regions of interest, a custom panel of Haloplex (Agilent technologies) was designed using the Agilent Sureselect website. **Supplementary Tables 4** and **5** provide the coordinates of amplicons and covered BED included in the sequencing panel, respectively.

### Targeted resequencing of genes frequently mutated in thymic epithelial tumors

**Figure 2:**
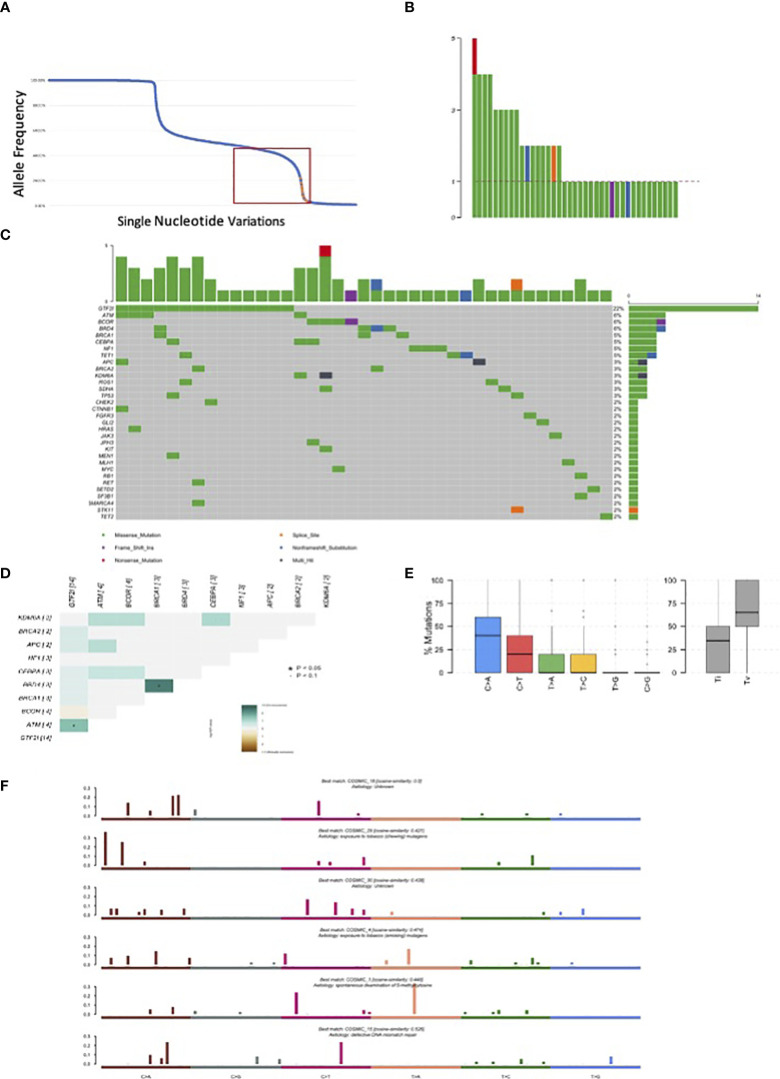
Mutations observed in our series of 67 resected thymomas associated to myasthenia gravis. **(A)** Orange dots represent GTF2I mutations; blue dots all other variations. The curve has a bimodal distribution with a relevant number of variations with an allele fraction of 100% or 50% corresponding to homozygous and heterozygous polymorphisms. **(B)** Number of candidate somatic mutations affecting the coding sequence observed for each tumor. **(C)** Most frequently mutated genes in thymomas and type of mutations observed according to color legend. **(D)** Concordance table of co-occurring and mutually exclusive mutations. Significant co-occurrence are highlighted with * if p<0.05 and with * if border line significant with p<0.1. **(E)** Boxplot representation of mutations observed in the coding sequence of thymomas. **(F)** Mutational signatures enriched in thymomas.

We sequenced 67 thymomas with myasthenia gravis, and the patients’ characteristics are summarized in **Table 2**. GTF2I mutations, which are somatic and specific to thymomas, were used as an internal control to assess our ability to identify somatic mutations among germline alterations. The allele frequency of single nucleotide variations is shown in **Figure 2A**. Among the entire set of mutations, there is a bimodal distribution around 100% and 50% allele frequencies, representing bona fide homozygous and heterozygous single nucleotide polymorphisms, respectively. GTF2I mutations have an allele frequency ranging from 4% to 22% due to the dilution of non-tumoral thymocytes and stromal cells within thymoma samples. We considered candidate somatic mutations to be those with an allele frequency between 45% and 4%.

**Table 2:** Patients’ characteristics.

Median Age (range)	55 years (26-83)
Male	33
Female	34
Histotype WHO	
A	12
AB	12
B1	5
B2	21
B2-B3	5
B3	8
Unknown	4
Stage Masaoka and Koga	
I	6
IIA	16
IIB	26
III	6
IVA	9
Unknown	4
GTF2I mutations	14
Myasthenia Gravis (Osserman’s classification)	
I	7
IIA	11
IIB	32
IIIA	4
IIIB	9
IVA	2
IVB	1
V	1

There were 238 mutations, of which 83 were in the coding sequence, including 65 missense, one non-sense, 13 silent mutations, and one frameshift insertion (**Table 3**, **Supplementary Table 7**). Among the thymomas, 43 had at least one mutation in the coding sequence. One patient had five mutations, three patients had four mutations, five patients had three mutations, and eight patients had two mutations (**Figure 2B**).

**Table 3:** Mutations of thymomas.

	Mutations	Mutations of the coding sequence
Samples with mutations	64	43
Genes with mutations	52	37
3’Flank	0	0
3’Untranslated Regions	6	0
5’Flank	0	0
5’ Untranslated Regions	2	0
Frame Shift Deletions	0	0
Frame Shift Insertions	1	1
In Frame Deletions	0	0
In Frame Insertions	0	0
Intergenic	13	0
Intron	132	0
Missense Mutation	65	65
Noncoding RNA	2	0
Non frameshift Substitution	2	2
Nonsense Mutation	1	1
Nonstop Mutation	0	0
Splice Site	1	1
Synonymous Mutation	13	13
***total***	*238*	*83*

There were 14 GTF2I mutations distributed according to histology: six in A thymomas, five in AB thymomas, two in B2 thymomas, and one in a thymoma with an unspecified subtype. *BRD4*, *ATM*, and *BCOR* were mutated in four samples (**Figure 2C**). A concordance table of non-synonymous somatic mutations was created (**Figure 2D**). *BRD4* and *BRCA1* mutations significantly co-occurred in the same tumors, while *ATM* and *GTF2I* mutations co-occurred in others. A trend for mutually exclusive events was observed for GTF2I and *BCOR* mutations. *BCOR* mutations consisted of a frameshift insertion in a B3 thymoma and missense substitutions in two B2 and one A TETs. Interestingly, the thymoma diagnosed as an A histotype did not harbor a *GTF2I* mutation and exhibited invasive growth into the mediastinal fat. *TP53* mutations were present only in two samples, one B2 and one B2/B3 thymoma. There were 9 patients with pleural or pericardial metastases at the diagnosis; none of them had GTF2I mutations. All the GTF2I mutated tumors were diagnosed in stage I or II. On the contrary 40% of tumors with BCOR mutation had metastases at the diagnosis. None of the patients with TP53 or HRAS mutations had metastases at the diagnosis.

The most common point mutations identified were C>A transversions and C>T transitions (**Figure 2E**). We identified six mutational signatures (**Figure 2F**), including COSMIC 1, which is associated with spontaneous deamination of 5’-methylcytosine. There were also two signatures related to tobacco carcinogenesis through chewing and smoking exposure (COSMIC29 and 4, respectively), COSMIC15 associated with DNA mismatch repair, and two unknown signatures (COSMIC18 and 30).

**Figure 3:**
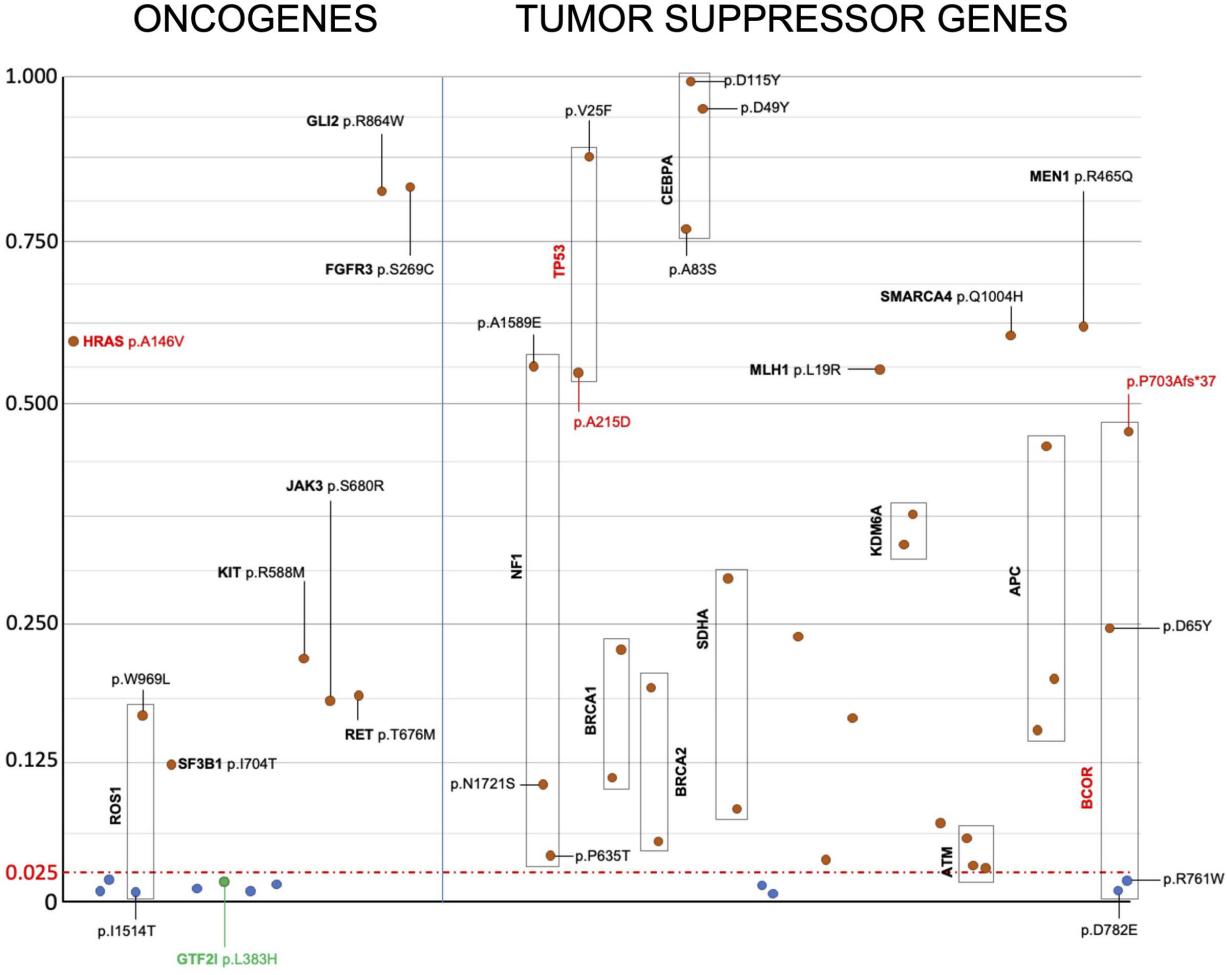
Prediction of the effect of candidate somatic mutations on the structure and function of oncogenes and tumor suppressor genes’ proteins. Candidate somatic mutations within the coding sequence, having a M-Cap score higher than 0.025, are predicted to alter protein structure or function and are reported in red. Mutations without any effect on protein structure or function, as predicted, are reported in blue. Despite being predicted to not alter protein function based on the M-Cap score, the GTF2I mutation is highlighted in green because it is known to drive cancer growth in TETs. Y axis indicate m-CAP score and x axis the mutations arbitrarily ordered.

Apart from *GTF2I*, we were unable to identify mutations present in the same codon as those reported in the literature (**Supplementary Table 2**). Therefore, there were no recurrent mutations identical to those previously reported. For tumor suppressor genes, various types of mutations can inactivate protein function and have a similar effect promoting tumor growth. In the case of oncogenes, specific mutations are known to confer the gain of function necessary to transform a proto-oncogene into an oncogene. According to the OncoKB database, only *GTF2I* L424H, *HRAS* A146V, *TP53* A215D, and *BCOR* P703Afs*37 are mutations capable of driving tumor growth (37). To understand the effect of the identified mutations, we predicted their impact on the structure and function of oncogenes and tumor suppressor genes by calculating the Mendelian Clinically Applicable Pathogenicity (M-CAP) Score (**Figure 3**). Mutations with an M-CAP score higher than 0.025 are predicted to affect protein function or structure. Tumor suppressor genes exhibited more mutations with a significant M-CAP score. Among the oncogenes, the *HRAS* A146V mutation had a significant score, while GTF2I L424H did not. Other mutations in oncogenes with a significant M-CAP score included *ROS1* W969L, *KIT* R588M, *JAK3* S680R, *RET* T676M, *SF3B1* I704T, *FGFR3* S269C, and *GLI* R864W.

We successfully identified mutations in the coding sequence of 36 tumors (54%), and our detection rate depended on the TET histotype (Fisher’s exact test p=0.048, combining B2-B3 with B3 tumors). Mutations in the coding sequence were observed in 83% of A thymomas and 20% of B2-B3 thymomas (**Table 4**).

**Table 4:** Coding sequence mutations in TET histotypes.

	A	AB	B1	B2	B2-B3	B3	NOS
CDS MUT	10	8	2	11	1	3	3
NO	2	5	3	10	4	10	1
Frequency	83%	62%	40%	52%	23%		

NOS, not otherwise specified; CDS MUT, coding sequence mutations.

## Discussion

Reviewing the results of whole genome, exome and targeted sequencing of TETs, we identified 4208 mutations in 339 patients and defined a panel of 63 genes frequently mutated in these tumors. We designed a targeted resequencing assay using Haloplex technology from Agilent and sequenced 67 thymomas resected from patients with myasthenia gravis. The only recurrent mutation observed in our analysis and in the reviewed literature was the missense L424H *GTF2I* mutation. There were no other oncogenes with recurrent mutations, while several tumor suppressor genes were predicted to be inactivated by the identified mutations.

TETs exhibit divergent clinical behavior: while some tumors grow as an expansive mass, others infiltrate the mediastinal fat and the surrounding structures or metastasize mostly into the pleural cavity (2). Moreover, some thymomas are associated with paraneoplastic syndromes, including myasthenia gravis (40%), Aplastic Anemia, and Hypogammaglobulinemia This association is likely due to impaired negative selection of thymocytes that recognize self-antigens in the aberrant thymus (2). Somatic mutations, copy number aberrations, and mRNA expression correlate with different kinds of TETs and help to differentiate tumors with different clinical features (9). TETs have one of the lowest tumor mutation burden observed in solid tumors, with an average of 0.48 mutations per mega-base (4). However, thymic carcinomas have a significantly higher number of mutations compared to thymomas (9). The most common mutation observed is the missense L424H mutation in GTF2I, which is unique to this type of neoplasm (38). Therefore, we decided to design a panel of genes for targeted resequencing specific to TETs. In 2018, we reviewed the literature identifying 4208 somatic mutations in 339 TETs, selecting those genes with recurrent mutations. Since then, additional reports of targeted resequencing of TETs evaluating common cancer genes have been published, confirming previous results (18, 21, 39, 40). Consequently, our current analysis remains updated to define the most relevant mutations in thymomas. The low mutation burden detected in thymomas has been attributed to the low number of somatic mutations in the DNA of tumor cells. However, this may be underestimated in some tumors where non-tumoral thymocytes outnumber cancer cells. This is frequent in AB, B1 and B2 histotypes where the number of tumor cells could be less than 10% of those present in the sample (2, 41). It is not surprising that mutations cannot be identified in some thymomas. Targeted resequencing can help identify some of these mutations, especially when a deep coverage is obtained. A targeted resequencing approach limited to the genes of interest can help achieve deep coverage, which is useful for detecting mutations in samples rich in non-neoplastic cells. Using the Halopex custom panel, we obtained a mean coverage of 1248 reads (average of the mean coverage of all samples; range 353-2542). However, after confirming the data with the Illumina Enrichment custom kit, we noticed that mutation calls with an allele fraction below 4% and a minimum coverage of 20 reads had a poor concordance between the two platforms. This could represent a limitation in detecting somatic mutations in samples rich in thymocytes and non-neoplastic cells of tumor microenvironment.

*GTF2I* mutation was the most common, present in 21% of the analyzed TETs, with 50% found in A thymomas and 38% in AB thymomas. This appears to be an underestimation compared to previous findings of 82% and 100% in A thymomas and 74% and 70% in AB thymomas reported by Petrini I et al. (9) and Radovich M. et al. (4), respectively. In the present evaluation, only patients with myasthenia gravis were included, which may introduce a selection bias compared to previous reports. Indeed, thymoma-associated myasthenia gravis is more common in B thymomas than A and AB histotypes and is absent in thymic carcinomas and micronodular thymomas (2, 4, 42). Moreover, in the present evaluation, tumor specimens were sampled from the resected mass and immediately frozen, potentially increasing the detection rate of GTF2I mutations using highly sensitive technologies. Using PyClone analysis, the presence of GTF2I mutation was observed in up to 64% (14/22) of thymomas including in those with B histotype (43%; 6/14) (43).

The missense mutation of GTF2I on chromosome 7:74146970 leads to a leucine-to-histidine substitution in the second conserved TFII-I repeat domain of the protein, near to the DNA binding site. There are six known isoforms of GTF2I, but thymomas express only isoforms 2 (Beta) and 4 (Delta), according to RNA sequencing results (9). L424H mutated GTF2I is an oncogene. The CRISPR/Cas9 knock-in of Gtf2i mutation leads to neoplastic transformation of murine immortalized thymic epithelial cells, which acquire the ability to form tumors when transplanted under the skin of nude mice (44). Moreover, two different mouse models expressing mutated Gtf2i in the thymus have been generated by two independent groups (45, 46). He Y. et al. generated a mouse model in which the expression of wild type Gtf2i is switched to L424H mutant, in the presence of Cre recombinase under the control of its own promoter. Subsequently, *Gtf2i* L424H-floxed mice were crossed with *Foxn1*-Cre mice to obtain Gtf2i L424H conditional KI mice, in which mutant *Gtf2i* L424H is expressed in Foxn1-expressing TECs (46). Giorgetti O.B. et al. generated a transgenic mouse model by inoculating a vector containing the mutant delta *Gtf2i* isoform under *Foxn1* promoter into pronuclei of fertilized mouse eggs (45). In both cases, young mice showed defects of thymic medulla development and maturation of medullary epithelial cells, while aged mice developed thymomas. Histologically, thymomas mirror human type B1 and B2 histotypes. The evaluation of the transcriptome of thymoma cells in transgenic mice showed a dysregulated expression of the genes associated with cortical, medullary and progenitor differentiation. According to TCGA data, human TETs with the *GTF2I* L424H mutation had an enrichment in cortical and intertypical thymic epithelial cell signatures compared with the *GTF2I* WT tumors (**Supplementary Figure S2**), which mirrors thymomas in the *Gtf2i* mutated mouse model. Differences between the mouse model and human tumors are expected. In human tumors, the somatic mutation of *GTF2I* is acquired in adult cells, whereas in mouse models, the mutation is already expressed during embryonal development of the thymus. In normal mice, *Gtf2i* and *Foxn1* follow a similar pattern of expression. Gtf2i and Foxn1 are highly expressed in early precursor cells and are subsequently more expressed in cortical than in medullary epithelial cells (45). Therefore, it is not surprising that transgenic mouse models expressing mutated Gtf2i under the promoter of *Foxn1* develop thymomas with cortical features instead of medullary features. Experiments using murine epithelial cells transformed by the CRISPER/Cas9 knock-in of *Gtf2i* mutation suggest enhanced resistance to apoptosis, increased resistance to DNA damage, and the ability to grow under stress conditions such as glucose deprivation (44). However, it remains to be elucidated how *GTF2I* mutation drives the neoplastic transformation of thymic epithelial cells suggesting that these novel murine models harboring *Gtf2i* L424H mutation represent an ideal tool for further studies.

Copy number gain and loss of 7q11.23, a cytoband containing the locus of GTF2I, are associated with Williams-Beuren and with 7q-microduplication syndromes two conditions with a symmetrically opposite phenotype. (PMID: 25501393) None of the patients with thymoma included in our evaluation had the diagnosis or symptoms suggesting these conditions.

Reviewing mutations described in the literature, we observed at least two groups of TETs with mutually exclusive mutations: one with *GTF2I* and another with *TP53* mutations. *GTF2I* mutations co-occurred with those of *HRAS*, *TTN*, and *UNC93B1* in the same group of tumors. On the contrary, mutations of *BRCA2*, *SETD2*, *PBRM1*, and *CDKN2A* significantly co-occurred with *TP53* mutation. In our series of thymomas, we did not observe the division in these two groups, probably because of the absence of thymic carcinomas. In our cases of thymomas from patients with myasthenia gravis, we identified a trend for the presence of mutually exclusive mutations of *GTF2I* and *BCOR*. *BCOR* is a tumor suppressor gene that encodes for an epigenetic regulator involved in the specification of cell differentiation and body structure development and takes part in the non-canonical polycomb repressive complex 1 (47). *BCOR* inactivating mutations have been previously described in thymomas (17) and in other kinds of tumors, including sarcomas (47) and lymphomas (48). In TCGA report, integrated analysis of CNV, mRNA, miRNA, DNA methylation, and reverse phase protein array identified four clusters of TETs (4). Subtype 1 is primarily represented by type B, subtype 2 by type TC, subtype 3 is primarily type AB, and subtype 4 is a mix of types A and AB. As expected, subtype 1 (mostly type B) was greatly enriched for cases with myasthenia gravis. Cases in subtypes 1 and 3 were associated with higher lymphocyte content, whereas *GTF2I* mutation was predominantly seen in subtypes 3 and 4. It remains to be understood if the presence of these clusters of gene expression is sustained by just two or more pattern of genomic mutations, whose detection remains challenging in tumors rich of lymphocytes. Enrichment of tumor cells in combination with deep coverage of sequencing could contribute to elucidate the somatic mutations of thymomas rich in non-neoplastic thymocytes.

In the current evaluation, C to A transversions were more common, whereas, according to the literature, C to T transitions were more frequently observed in TETs. Different mutation patterns have been reported for different types of tumors. For example, lung cancers share a mutation spectrum dominated by C to A mutations, consistent with their exposure to the polycyclic aromatic hydrocarbons in tobacco smoke, whereas melanomas show frequent C to T mutations caused by an altered repair of ultraviolet-induced covalent bonds between adjacent pyrimidines (49). Since mutations are more common in thymic carcinomas, the high incidence of C to T transitions reported in the literature could be related to these kinds of TETs. On the contrary, our analysis includes only thymomas with myasthenia gravis and possibly a different pattern of mutations sustained by a different etiological factor can be supposed for these tumors. In the literature, COSIMIC 1 mutational signature (spontaneous deamination of 5-methylcysteine) was observed in *GTF2I* mutant and wild-type tumors. On the contrary, COSMIC 5 (unknown etiology) was observed in *GTF2I* tumors and COSMIC 6 (defective mismatch repair) in *TP53* mutated tumors. In the current series, the presence of COSMIC 1 signature and of a signature associated to mismatch repair defect was observed (COSIMC 15). COSMIC 1 signature is associated to spontaneous deamination of 5-methylcysteine, a phenomenon observed in aging. This observation is concordant with the biology of TETs that usually occurs in older people. Mutational signatures could suggest a similar etiology for the initial transformation of thymic epithelial cells, but subsequent different routes of mutations in *GFT2I* mutated and in *TP3* mutated TETs.

Several targeted therapies have been tested for the treatment of advanced TETs with limited clinical efficacy. *GTF2I* was the only recurrent mutation between the literature and the current sequencing analysis. To date, there is no drug able to inhibit mutated GTF2I. Moreover, *GTF2I* mutated tumors usually present an indolent clinical behavior and surgery is curative for most of the cases. In order to identify possible targets for therapies, we used the M-CAP score to predict the effect of the observed mutations on oncogenes and tumor suppressor gene function. M-CAP is a classifier of somatic mutations that combines the results of multiple algorithms in a score with high sensitivity in predicting the chance of a functional or a structural aberration of the protein. Among oncogenes, *HRAS* A146V, *ROS1* W969L, *KIT* R588M, *JAK3* S680R, *RET* T676M, *SF3B1* I704T, *FGFR3* S269C and *GLI* R864W had significant M-CAP scores. ROS1, RET, FGFR3 and KIT are tyrosine kinase receptors of growth factors and JAK3 is a tyrosine kinase associated to growth factor receptors. Different inhibitors are available for each one of these genes, but the episodical observation in few samples limits our chance to design a clinical trial for a specific target. Moreover, the mutation should confer a gain of function of the protein in order to transform a proto-oncogene into an oncogene. None of these tyrosine kinase mutations has been reported to be oncogenic in the literature. The mutation of *FGFR3* S269C can activate the receptor by adding a cysteine residue in its extracellular domain implicated with altered skeletal formation when germline but not with the formation of tumors. *FGFR3* is considered an oncogene but has also tumor-suppressive properties in cells with epithelial phenotype (50). Only *HRAS* A146V has been previously described in colorectal cancers, and it is possibly implicated in tumorigenesis (51). However, the *HRAS* mutation observed is not the classical mutation of codon G12 or G13, which confers a slower hydrolysis of GTP. Specific inhibitors are available for the G12C mutation (52). Interestingly, the *HRAS* A146V co-occurred with *GTF2I* L424H mutation as predicted by the concordance table of the mutations observed in the literature. Tumor suppressor genes can be inactivated by several different mutations and multiple genes. We observed three inactivating mutations of *NF1*, supporting the importance of RAS activation for the development of TETs. NF1 functions as GAP protein, enhancing the hydrolysis of GTP bounds to RAS to GDP and switching off the downstream transduction of the intracellular signal pathway. Targeted therapies for inactivated tumor suppressor genes are not available to date and specific targets for aggressive thymomas remain to be identified for effective treatment.

From a clinical perspective, our analysis did not identify any mutations immediately eligible for targeted treatment. However, it does suggest a molecular subclassification of thymomas into at least two subgroups. We have confirmed that GTF2I mutated tumors exhibit a better prognosis because diagnosed only in stage I-II. Conversely, the other group of thymomas demonstrates a worse prognosis, and although a highly prevalent driver mutation has not been identified yet, BCOR mutations appear to be enriched within this subgroup. Due to the low incidence of thymomas, single-arm phase II trials are typically conducted to assess the efficacy of novel drugs. In order to better contextualize the results of these trials, it is important to include a prediction of the clinical behavior of thymomas based on their molecular aberrations. Our designed panel of genes could serve as a useful tool for the molecular classification of thymic epithelial tumors. This is particularly valuable because some of the most interesting genes mutated in thymomas are not included in standard panels for next-generation sequencing.

In conclusion, based on a comprehensive review of the literature, we have developed a gene panel for targeted resequencing of thymic epithelial tumors and confirmed the presence of GTF2I mutations in a subgroup of thymomas among patients with myasthenia gravis.

## Data availability statement

The original contributions presented in the study are included in the article/supplementary material, further inquiries can be directed to the corresponding author/s.

## Ethics statement

The studies involving human participants were reviewed and approved by Ethics Committee of Tuscany Region for Clinical Trials - Section of North West Area (CEAVNO). The patients/participants provided their written informed consent to participate in this study.

## Author contributions

EP: Methodology, Investigation; FCu: Software, Investigation, Writing - Review & Editing; SP: Writing - Review & Editing; GT: Methodology; AV: Investigation; FCo: Resources, Writing - Review & Editing, Funding acquisition; VN: Resources; MG: Investigation, Writing - Review & Editing; MM: Resources; RR: Supervision; VA: Resources; MA: Resources; SB: Investigation, ML: Resources, Supervision; IP: Methodology, Writing - Original Draft, Supervision, Funding acquisition. All authors contributed to the article and approved the submitted version.
