# Supplementary material for: Somatic mutations of thymic epithelial tumors with myasthenia gravis
Source: Front Oncol. 2023 Aug 21;13:1224491. doi: 10.3389/fonc.2023.1224491 (PMC10475716; doi:10.3389/fonc.2023.1224491)
Supplement: Supplementary file 1 [file DataSheet_1.docx]

Supplementary Material

Somatic mutations of thymic epithelial tumors with myasthenia gravis

Eleonora Pardini, Federico Cucchiara, Sara Palumbo, Giulia Tarrini, Alessia Di Vita, Fabio Coppedè, Vanessa Nicoli, Melania Guida, Michelangelo Maestri, Roberta Ricciardi, Vittorio Aprile, Marcello C. Ambrogi, Serena Barachini, Marco Lucchi and Iacopo Petrini*

*** Correspondence:**Iacopo Petrini MD, PhD

Department of Translational Research and of New Surgical and Medical Technologies,

University of Pisa, Italy

Via Savi 2, Pisa, 56121, Italy

00390502212571, FAX 0039050 993378

iacopo.petrini@unipi.it

**Supplementary Figures:** According to the literature, review data are divided in TETs with and without GTF2I mutation.

**Supplementary Figure S1:** Type of mutations in TETs with GTF2I mutation: missense mutations were the most common; single nucleotide variations (SNP) were more common than insertion (INS) and deletions (DEL). C to T transition were the most common in TETs with GTF2I mutation. The second most frequent mutations after GTF2I were those of HRAS.

**Supplementary Figure S2:** Type of mutations in TETs without GTF2I mutation (S1C): missense mutations were the most common; single nucleotide variations (SNP) were more common than insertions (INS) and deletions (DEL). C to T transition were the most common in TETs without GTF2I mutations. Tp53 mutation was the most common followed by those of SETD2, ATM, CDKN2A, PBRM1, HRAS and APC.

**Supplementary Figure S3:** COSMIC1 (spontaneous deamination of 5-methylcytosine) and COSMIC5 (unknown etiology) signatures were enriched in TETs with GTF2I mutation.

**Supplementary Figure S4:** COSMIC1 (spontaneous deamination of 5-methylcytosine) and COSMIC6 (defective DNA mismatch repair) signatures were enriched in TETs without GTF2I mutation.
